# Hidradenitis supparativa complicated by penoscrotal lymphedema and renal amyloidosis

**DOI:** 10.15537/smj.2022.43.7.20220352

**Published:** 2022-07

**Authors:** Nouf F. Bin Rubaian, Haya F. Al Zamami, Seereen R. Almuhaidib, Sarah H. Al Breiki

**Affiliations:** *From the Department of Dermatology (Bin Rubaian, Al Zamami, Almuhaidib), College of Medicine, Imam Abdulrahman bin Faisal University, Dammam, and from the Department of Dermatology (Al Breiki), King Fahad Hospital of the University, Khobar, Kingdom of Saudi Arabia.*

**Keywords:** hidradenitis suppurativa, lymphedema, amyloidosis

## Abstract

Genital lymphedema usually develops after 4-30 years of chronic hidradenitis suppurativa (HS). However, our patient exhibited signs of it as early as 2 years after being diagnosed with HS. Renal amyloidosis is a rarely reported complication of HS. Unfortunately, our patient was asymptomatic but was found to have end-stage renal disease secondary to advanced renal amyloidosis. We report a case of a 42-year-old Indian gentleman who had HS for 9 years presenting with 2 rare complications: penoscrotal lymphedema and end-stage renal disease secondary to renal amyloidosis. The patient was treated with prednisolone and adalimumab to treat both his HS and renal amyloidosis, and was referred to general surgery to manage his genital lymphedema. We recommend following adult patients with moderate-to-severe HS and clinical duration of greater than 3 years and screening for amyloidosis before they reach end-stage organ disease, similar to what happened to our patient with end-stage renal disease.

**H**idradenitis suppurativa (HS) is a disease affecting the follicular epithelium, causing obstruction of the follicle with secondary apocrine gland involvement, characterized by painful recurrent pustules, nodules, abscesses, and sinus tract formation, involving mainly the axillae, groin, buttocks, and infra-mammary regions.^1^ It causes significant quality of life impairment.^2,3^ Hidradenitis suppurativa is associated with many cutaneous and systemic complications, such as contracture, scaring, anemia, arthropathy, lymphedema, squamous cell carcinoma, and amyloid A (AA) amyloidosis.^1,2^

Lymphedema is a complication of severe and long-standing HS that results from lymphatic drainage obstruction, resulting in chronic inflammation.^3^ The most commonly affected area is the genitals, presenting clinically as soft tissue swelling, ranging from mild swelling to scrotal elephantiasis.^2^ Genital lymphedema may cause further debilitation by negatively impacting sexual and urological functions, and aesthetic appearance.^3^

Amyloid A amyloidosis is a progressive multi-organ disease; it is a rare complication of HS.^1^ It is caused by the overproduction and deposition of serum AA in the tissues, damaging the affected organ.^1^ Although it most commonly presents with kidney involvement, it can affect other organ systems as well, such as the cardiovascular, gastrointestinal, and musculoskeletal systems.^1^ In severe and untreated cases, renal AA amyloidosis may progress into renal failure and cause death.^1^

## Case Report

**Figure 1:**
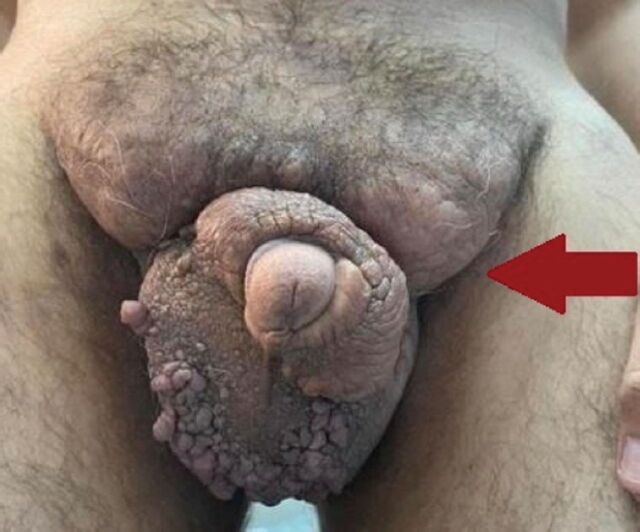
- Penoscrotal lymphedema.

A 42-year-old Indian gentleman, non-smoker, with a body mass index of 24 kg/m^2^ presented with a history of HS of the axillary area for 9 years. Two years after axillary HS, he developed HS over the perineal area. Progressive swelling and multiple asymptomatic non-draining skin-colored papules and nodules were noted; some of them were pedunculated, involving the inguinal area, penile shaft, scrotum, and perineum (Figure 1).

### Diagnostic assessment

Over the years, he had been treated with topical and oral antibiotics and oral acitretin. As the condition did not show marked improvement, starting adalimumab was considered. Upon further investigation, he was found to have latent tuberculosis, for which he completed a full anti-tuberculosis regimen.

### Therapeutic intervention

In December 2018, adalimumab was initiated at 40 mg subcutaneously once a week for 4 months, then discontinued as he had a risk of tuberculosis reactivation due to multiple spikes of fever. Therefore, he was shifted to oral acitretin. After several months, a purified protein derivative test was negative, so adalimumab was started again in September 2019 with an induction dose of 160 mg at baseline and 80 mg after 2 weeks, followed by a standard dose of 40 mg subcutaneously once a week. After 4 months, multiple episodes of low-grade fever spikes occurred, for which adalimumab was discontinued again. Since then, he has not taken any medications for his HS condition.

### Follow-up and outcomes

**Figure 2:**
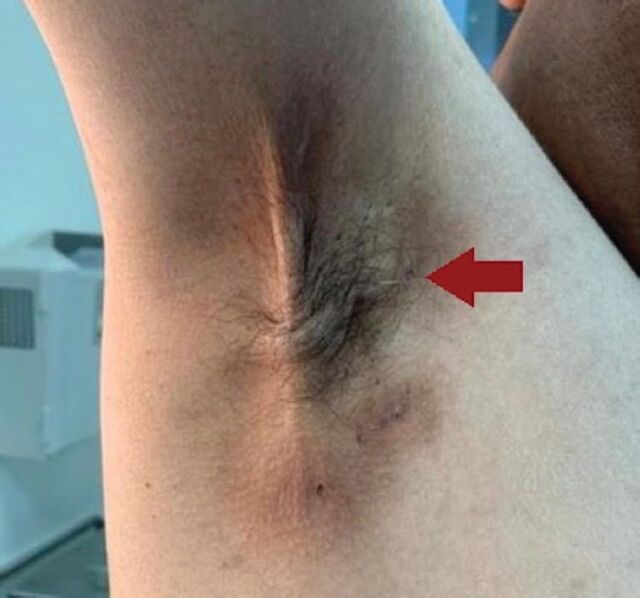
- Mild hidradenitis supparativa with only post inflammatory hyperpigmentations. He gets occasional small nodules that get resolved with topical treatment.

**Figure 3:**
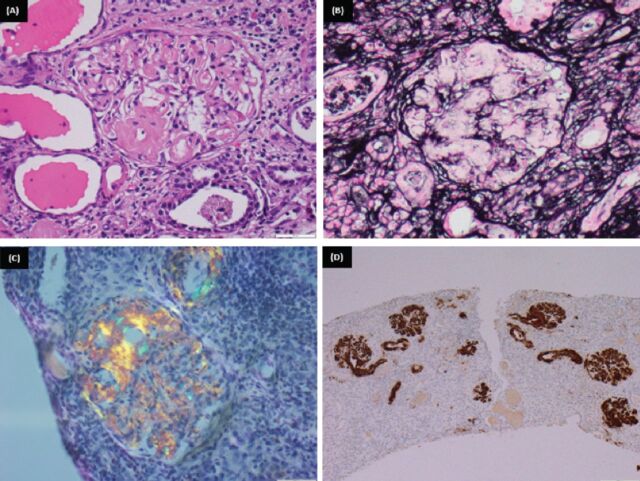
- A glomerulus shows: A) abundant mesangial as well as capillary wall deposits of amorphous eosinophilic material (H&E - x400). B) The deposited material is silver negative (JMS-x400). C) Congo red stain revealed apple green birefringence under polarized light (Congo red-x400). D) The tufts are strongly positive for amyloid associated protein (immunoperoxidase, AA protein-x400).

During the pandemic, he failed to follow up at the dermatology clinic and was no longer receiving treatment. In August 2020, he returned for follow up with a few inflammatory nodules on the axillae (Figure 2). At that time, his renal function showed elevated serum creatinine and urine protein, and a biopsy was carried out. He was diagnosed with stage V chronic kidney disease secondary to advanced renal amyloidosis with interstitial nephritis (Figure 3). He was treated with prednisolone, and reintroduction of adalimumab was considered to treat both his HS and renal amyloidosis. He was also referred to general surgery to manage his genital lymphedema (Table 1).

**Table 1:** - Patient’s summarized timeline.

Dates	Relevant past medical history and interventions	
	Diagnostic testing	Intervention
March 2017	Adalimumab was considered, but latent tuberculosis was detected	Full anti-tuberculosis regimen
December 2018	Purified protein derivative test was negative	Adalimumab was initiated
January 2020	-	Adalimumab was discontinued due to reoccurring spikes of fever
August 2020 (At the time of patient presentation)	Renal function test showed elevated serum creatinine and urine protein. Renal biopsy showed stage V chronic kidney disease secondary to advanced renal amyloidosis	Prednisolone, and reintroduction of adalimumab was considered to treat both his HS and renal amyloidosis. The patient was also referred to general surgery to manage his genital lymphedema.

HS: hidradenitis suppurativa

## Discussion

Hidradenitis suppurativa, also known as acne inversa, is a chronic inflammatory cutaneous disease affecting apocrine gland bearing areas.^1^ Lymphedema is a well-described complication of HS affecting more males than females. It involves the scrotum, penis, labia majora, perineum, groin, buttocks, and abdomen.^2^ Penoscrotal lymphedema is the most common presentation of lymphedema secondary to HS.^2^ Furthermore, genital lymphedema usually develops after 4-30 years of chronic HS.^2^ However, our patient exhibited signs of it as early as 2 years after being diagnosed with HS.

Lymphedema in HS presents as polypoid verrucous papules or nodules which may range from mild swelling to scrotal elephantiasis.^2^ In the advanced stages, the lesions can be clinically indistinguishable from verrucous carcinoma, so they need to be biopsied.^2^ In addition, chronic HS patients have a 50% increased risk of any type of malignancy and a squamous cell carcinoma risk of 4.6%.^4,5^

The cornerstone treatment for lymphedema is physical compression therapy; other medical options are infliximab, adalimumab, minocycline, and isotretinoin.^2,3^ However, limited evidence supports the efficacy of managing lymphedema solely medically.^4^ Furthermore, the only curative management is surgical removal.^2,3^ Patients who underwent surgical management reported an improvement in quality of life and a full or adequate return of sexual function.^6^

Although AA amyloidosis is a well-recognized complication of chronic inflammatory diseases, it has rarely been reported in HS.^1^ Uncontrolled inflammation causes an overproduction of serum AA, an acute-phase reactant protein, resulting in tissue deposition and functional interference.^1^ The most common site of involvement is the kidneys, presenting as asymptomatic proteinuria or nephrotic syndrome that may progress to renal failure.^1,7^ The recent literature showed that around 45% of patients with renal AA amyloidosis secondary to HS developed renal failure.^7^ It presents in patients with ages ranging between 37-73 years and follows HS onset by 3-51 years.^7^ This is consistent with our patient, who was 42 years old and had an HS duration of 9 years. He presented with nephrotic proteinuria, and the diagnosis of renal amyloidosis was confirmed by biopsy.

The core management of AA amyloidosis is to control the underlying inflammatory disease, which will result in the stabilization or even regression of amyloid deposition.^8^ In severe cases of HS, periodic screening with serum creatinine, urea, electrolytes, and urine protein should be maintained.^1^

In conclusion, we recommend following adult patients with moderate-to-severe HS and a clinical duration of greater than 3 years and screening for amyloidosis before they reach end-stage organ disease.

## References

[1] Girouard SD , Falk RH , Rennke HG , Merola JF. Hidradenitis suppurativa resulting in systemic amyloid A amyloidosis: a case report and review of the literature. Dermatol Online J 2012; 18: 2. 22301039

[2] Micieli R , Alavi A. Lymphedema in patients with hidradenitis suppurativa: a systematic review of published literature. Int J Dermatol 2018; 57: 1471–1480. 30105858 10.1111/ijd.14173

[3] Kridin K , Amber KT , Comaneshter D , Cohen AD. Amyloidosis in hidradenitis suppurativa: a cross-sectional study and review of the literature. Clin Exp Dermatol 2020; 45: 565–571. 31989656 10.1111/ced.14186

[4] Lapins J , Ye W , Nyrén O , Emtestam L. Incidence of cancer among patients with hidradenitis suppurativa. Arch Dermatol 2001; 137: 730–734. 11405761

[5] Chapman S , Delgadillo D III , Barber C , Khachemoune A. Cutaneous squamous cell carcinoma complicating hidradenitis suppurativa: a review of the prevalence, pathogenesis, and treatment of this dreaded complication. Acta Dermatovenerol Alp Pannonica Adriat 2018; 27: 25–28. 29589641

[6] Chen ML , Odom B , Santucci RA. Surgical management of genitoperineal hidradenitis suppurativa in men. Urology 2014; 83: 1412–1417. 24685061 10.1016/j.urology.2014.01.011

[7] Real de Asúa D , Costa R , Galván JM , Filigheddu MT , Trujillo D , Cadiñanos J. Systemic AA amyloidosis: epidemiology, diagnosis, and management. Clin Epidemiol 2014; 6: 369–377. 25378951 10.2147/CLEP.S39981 PMC4218891

[8] Obici L , Merlini G. AA amyloidosis: basic knowledge, unmet needs and future treatments. Swiss Med Wkly 2012; 142: w13580. 22653707 10.4414/smw.2012.13580

